# The Principles of Treatment

**Published:** 1900-12

**Authors:** 


**The Principles of Treatment.** By J. Mitchell Bruce, M.D. Pp. xv., 614. Edinburgh: Young J. Pentland. 1899.

If somewhat tardily we notice Dr. Mitchell Bruce's book, it is in part because the unusually solid character of its contents has made the perusal of the entire book not speedily nor lightly accomplished. It is superfluous to say that Dr. Mitchell Bruce has written a good book on a subject of which he is an acknowledged master; but we confess that it seems to us that in some directions compression might have been beneficially exercised, and on the other hand some topics are omitted which should have been included. We presume also, from the absence in most sections of any very recent references, the book has been a considerable time in preparation. The first nine chapters are devoted to the consideration of the general principles of treatment, and contain a great deal that is sound, judicious and instructive—especially chapter vi.—but contain also a good deal of what is hardly above platitude. While, therefore, the book is a store of ripe experience, it does not call for any very minute analysis. We note, however, one or two points that struck us during the perusal. Indigestion, here synonymous with gastric catarrh, is treated at some length and fairly well, but not handled in the light of modern research into the chemistry of stomach function. In the chapter on cirrhosis of the liver no allusion is made to Rutherford Morison's operation in cases which Dr. Bruce admits to be otherwise hopeless. General peritonitis being treated of, there was no necessity for a separate consideration of appendicitis, which when once diagnosed should be handed over to the surgeon: a disease much more pertinent to the physician, viz., diphtheria, is omitted altogether. This we think is a mistake, considering how anxious a care every case is to the medical attendant and how difficult the treatment often is. No disease of the spinal cord is treated. The section on typhoid fever seems to have been written comparatively recently, containing some recent references. It gives the line of treatment at present accepted by most physicians. But it is the author's views on the treatment of phthisis which struck us more than anything else in the book as being distinctly belated. We will quote a few sentences to show what we mean. The phthisical subject "is recommended to leave a cold and damp country . . . because at home he would be driven into confined quarters." "Morphine . . . would make the patient sleep into the forenoon inhaling the polluted atmosphere of his bedroom." "We forbid unwise exposure to cold, damp, wind or dust, out-of-doors." "The subject of phthisis must retire to bed early . . . to escape the impure atmosphere of the sitting-room occupied by the family circle, the products of expiration and transpiration and of blazing gas, the odour of the last meal, and the tobacco smoke." "When phthisis has entered on its second stage it is already too late to propose movement from home . . . the best place for a patient with softening tubercle is home." "The times and manner of taking food must be revised. Excessive frequency and excessive amount must be temporarily avoided." There is no need to multiply these extracts; they indicate views now gone by, one would have thought. We find no allusion to the open-air method, either absolute or modified, as now carried out either in this country or abroad.

If we presume thus to cavil somewhat at this book, it is not that we do not recognise its considerable merit, but we cannot believe that it fairly represents Dr. Mitchell Bruce and his present practice.

